# Efficacy and safety of P_11_-4 for the treatment of periodontal defects in dogs

**DOI:** 10.1007/s00784-021-04297-6

**Published:** 2022-01-10

**Authors:** Claudine Bommer, Tobias Waller, Monika Hilbe, Daniel Wiedemeier, Nina Meyer, Stephanie Mathes, Ronald Jung

**Affiliations:** 1 Credentis ag, Dorfstrasse 69, 5210 Windisch, Switzerland; 2 Clinic for Reconstructive Dentistry, University of Zurich, Plattenstrasse 11, 8032 Zurich, Switzerland; 3 Laboratory for Animal Model Pathology (LAMP), Institute of Veterinary Pathology, University of Zurich, Winterthurerstrasse 268, 8057 Zurich, Switzerland; 4 Center of Dental Medicine, Statistical Services, University of Zurich, Plattenstrasse 11, 8032 Zurich, Switzerland; 5 Department for Chemistry and Biotechnology, Zurich University of Applied Sciences, 8820 Wädenswil, Switzerland

**Keywords:** Periodontal disease, Self-assembling peptide P11-4, Regenerative scaffold, Dogs, Dehiscence defects

## Abstract

**Objectives:**

This study’s aim was to investigate the safety and performance of a self-assembling peptide matrix (SAPM) P_11_-4 for the treatment of periodontal disease in a controlled pre-clinical study.

**Materials and methods:**

Acute buccal bony dehiscence defects (LxW: 5 × 3 mm) were surgically created on the distal root of four teeth on one mandible side of 7 beagle dogs followed by another identical surgery 8 weeks later on the contralateral side. SAPM P_11_-4 (with and without root conditioning with 24% EDTA (T1, T2)), Emdogain® (C) and a sham intervention (S) were randomly applied on the four defects at each time point. Four weeks after the second surgery and treatment, the animals were sacrificed, the mandibles measured by micro-computed tomography (µ-CT) and sections of the tissue were stained and evaluated histologically.

**Results:**

Clinically and histologically, no safety concerns or pathological issues due to the treatments were observed in any of the study groups at any time point. All groups showed overall similar results after 4 and 12 weeks of healing regarding new cementum, functionality of newly formed periodontal ligament and recovery of height and volume of the new alveolar bone and mineral density.

**Conclusion:**

A controlled clinical study in humans should be performed in a next step as no adverse effects or safety issues, which might affect clinical usage of the product, were observed.

**Clinical relevance:**

The synthetic SAPM P_11_-4 may offer an alternative to the animal-derived product Emdogain® in the future.

**Supplementary Information:**

The online version contains supplementary material available at 10.1007/s00784-021-04297-6.

## Introduction

A prospective candidate for the treatment of periodontal disease caused by inflammation in response to a dysbiotic shift in the oral biofilm [1–4] presents the family of rationally designed self-assembling peptides (SAPs) to support periodontium’s intrinsic regeneration potential. SAPs have recently become the focus of tissue engineering and are promising candidates for the regeneration of soft and hard tissue damaged by disease, injury or trauma [5, 6]. Scaffolds play a vital role for adhesion, migration, proliferation, differentiation of cells involved in the regeneration process as well as their ability to carry signalling molecules for mediating cellular responses [7]. The self-assembling peptide P_11_-4 consists of eleven naturally occurring amino acids with the primary structure of CH_3_CO-Gln-Gln-Arg-Phe-Glu-Trp-Glu-Phe-Glu-Gln-Gln-NH_2_ assembling under specific physico-chemical conditions to form β-sheets as secondary structure subsequently building fibres finally resulting in a synthetically produced SAP P_11_-4 matrix [5, 8, 9]. A hydrogel of SAP P_11_-4 matrix (SAPM P_11_-4) was recently reported to be a suitable scaffold for dental follicle stem cells facilitating their proliferation, osteogenic differentiation and collagen type I expression in vitro [10]. Favourable cellular interactions of the SAP P_11_-4 scaffold were also found with other periodontal cells such as human periodontal ligament fibroblasts and osteoblasts suggesting that a SAPM P_11_-4 hydrogel is an injectable, biocompatible and non-cytotoxic scaffold candidate for periodontal hard and soft tissue regeneration therapy. Moreover, the SAPM P_11_-4 mimics the natural extracellular matrix with appropriate enzymatic and bacterial degradation characteristics found in the oral cavity [11–13].

El-Sayed et al. [14] were the first to study SAPM P_11_-4 for the treatment of periodontal disease in vivo. Bilateral maxillary critical-sized periodontal defects were generated in 13 rats. On one side a hydrogel of 10 mg/mL SAPM P_11_-4 was applied, the contralateral defect was left untreated as control. Rats were sacrificed post-surgery at time points 0, 2 and 4 weeks. Both treated and untreated defects showed clear tissue regeneration at both time points compared to time 0. Histological analysis showed greater organisation of periodontal fibres in defects treated with SAPM P_11_-4, at both time points, compared to untreated defects where mainly the formation of epithelial tissue and non-functional collagen fibres were observed indicating repair of lost tooth structure rather than regeneration.

The first regenerative approach involving a matrix for the treatment of periodontal disease is the enamel matrix derivative (EMD), which was studied during the last 20 years in several in vitro, animal and human studies [15]. Its established product (Emdogain®, Straumann) is derived from the enamel layer of developing porcine teeth and is a heterogeneous mixture of proteins containing for example amelogenins. Amelogenin self-assembles into supramolecular aggregates that form an insoluble extracellular matrix [16] and EMD is considered the gold standard for the treatment of self-contained intrabony defects [17]. Animal-derived products have several disadvantages, for example their intrinsic heterogenicity and the potential to transfer diseases. Therefore, the ultimate aim in the development of new biomedical products is to substitute products of animal origin with a synthetic product having similar properties and comparable treatment effects.

To substantiate the promising data in rats, the present study was designed to raise first efficacy and safety data on SAPM P_11_-4 for the treatment of periodontal disease in a pre-clinical study in dogs. In terms of etiopathology, periodontal defects in dogs closely resemble periodontal disease in humans [18]. Acute buccal self-contained dehiscence-type bone defects were chosen as these moderate defects were considered the target indication for the potential SAPM P_11_-4 hydrogel under evaluation for application in the treatment of periodontal disease and the prevalence of moderate periodontitis is higher compared to more severe cases. We favoured the acute model as the burden for the dogs is higher in acute-chronic models due to additional surgeries and a longer experimental period resulting in increased resource needs [19, 20].

The objectives of this randomized, controlled study were performance and safety evaluation of an SAP P_11_-4 matrix (T1, T2) in comparison to a sham operated control (S) and the clinical gold standard (Emdogain®, C) in the treatment of acute buccal bony dehiscence defects in the lower jaws of dogs.

## Materials and methods

### Animals, ethical statement and study design

Seven healthy, experimentally naïve, male Beagle dogs (10–13 kg, ≥ 1-year old, source: Centre d'Elevage des Souches, France) were chosen for this study. The study was performed in compliance with the European Directive 2010/63 on the protection of animals used for scientific purposes, ISO 10993 Part 2 (2006) / Part 6 (2007) and Good Laboratory Practice regulations. Approval was obtained from the Ethical Committee by the accredited and registered facility NAMSA having an animal welfare body (Chasse-sur-Rhône, France) prior to study start.

**Fig. 1:**
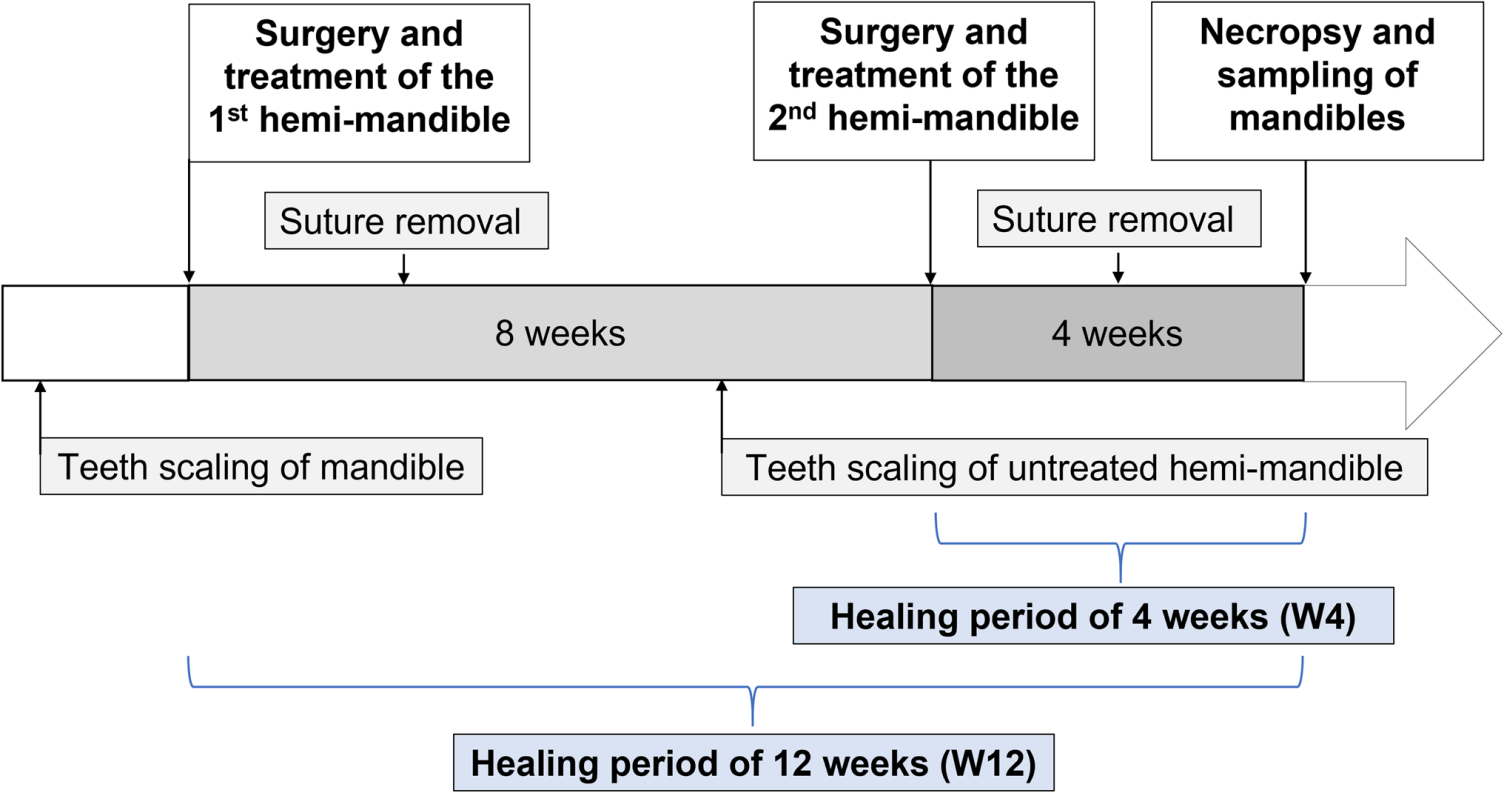
Trial design illustrating the schedule of procedures, surgeries and interventions for test and control lesions as well as the resulting healing periods

The animals were kept in the NAMSA facility 2 weeks before study start (first surgery) during which they were monitored. Animals were housed in groups in cages kept hygienic, environmentally enriched and of appropriate size allowing normal behaviour under laboratory conditions (room temperature of 15–21 °C, light cycles of 12 h in light resp. darkness, fed twice daily with soft food (SAFE, France) and clean water ad libitum). In total the dogs received two surgeries and treatments (Fig. 1): at the first surgery four acute buccal self-contained dehiscence-type bone defects were created on premolars P2, P3, P4 and molar M1 on one side of the mandible followed by another identical surgery 8 weeks later on the contralateral side. The dogs received a scaling of the teeth 10 days before the surgeries, the treatments on the surgery day and sutures were removed 14 days after each surgery. 12 weeks after the first surgery animals were sacrificed resulting in a healing period for one of the hemi-mandibles of 4 weeks (4 W) and for the other of 12 weeks (12 W). This design together with the creation of buccal dehiscence bony defects allowed to investigate four treatment groups at two different healing periods using as few dogs as possible for ethical reasons. In all, the safety was evaluated in 7 dogs and efficacy in four treatment groups for two time points with 8 study sites per dog resulting in totally 56 study sites: 7 sites per treatment and per healing period (4 weeks and 12 weeks).

### Preparation of SAPM P11-4 hydrogel, study groups, and treatments

In preparation of this study, usability of different SAPM P_11_-4 hydrogel concentrations and application systems were tested on pig jaws obtained from a regional slaughterhouse to identify the most suitable hydrogel and system regarding consistency, applicability, and handling. The used investigational product included a double chamber syringe system (Double Syringe, 2.5 ml, 1:1, PP‐R, Art. 103,501, Sulzer Mixpac, Haag, CH) where one chamber contained monomeric SAP P_11_-4 at 65 mg*ml-1 in a 0.04 M disodium hydrogen phosphate buffer and the other chamber contained 0.02 M citric acid resulting in an SAPM P_11_-4 hydrogel of 32.5 mg/mL in a disodium hydrogen phosphate—citrate buffer system. Sterilization (Leoni Studer AG, Däniken, CH) and sterility and bioburden testing (Labor Zollinger, Zurich, CH) was done prior to study start.

The following four study treatments were randomly applied to the root surface with full defect coverage during the first and second surgery after the creation of the bony defects:

1. Sham (S), sham operated control (periosteum coverage): denuded root surface was pre-conditioned with 24% EDTA (pH 7.0) for ~ 2 min and washed off with saline
2. Control (C), EMD gold standard control: 24% EDTA as described above and EMD application (Straumman Emdogain®) according to manufacturer’s instructions
3. Test1 (T1): SAPM P_11_-4 hydrogel (32.5 mg/mL) with 24% EDTA pre-treatment as described above
4. Test2 (T2): solely SAPM P_11_-4 hydrogel (32.5 mg/mL, no pre-conditioning of root surface)

Special care during the application was taken to avoid cross-contamination.

### Surgical and other procedures

Pre-operatively, the dogs received an injection of anti-inflammatory (carprofen, Rimadyl®, Zoetis) and analgesic drugs (medetomidine, Dorbene Vet®, Zoetis; buprenorphine, Buprecare®, Axience). Surgical procedures were performed under anaesthesia with intravenous injection of ketamine (Ketamine 1000®, Virbac) and locally using lidocaine with adrenaline (Lidocaïne adrenaline®, Aguettant). Each dog was laterally placed on a warmed pad, a neutral ophthalmic ointment (Ocrygel®, Laboratoire TVM) was applied on their eyes to protect the corneas. The dogs were intubated, mechanically ventilated, placed on isoflurane inhalant anaesthetic (Isoflo®, Axience) for continued general anaesthesia and received intravenous infusion with electrolyte solution (Ringer lactate, Baxter) during surgery. Temperature, electrocardiogram, peripheral non-invasive arterial blood pressure and oxygen saturation were monitored and when any abnormality was detected, appropriate clinical measures were taken. The mouth and surgical sites were sanitized with chlorhexidine (Cooper) soaked gauze.

**Fig. 2:**
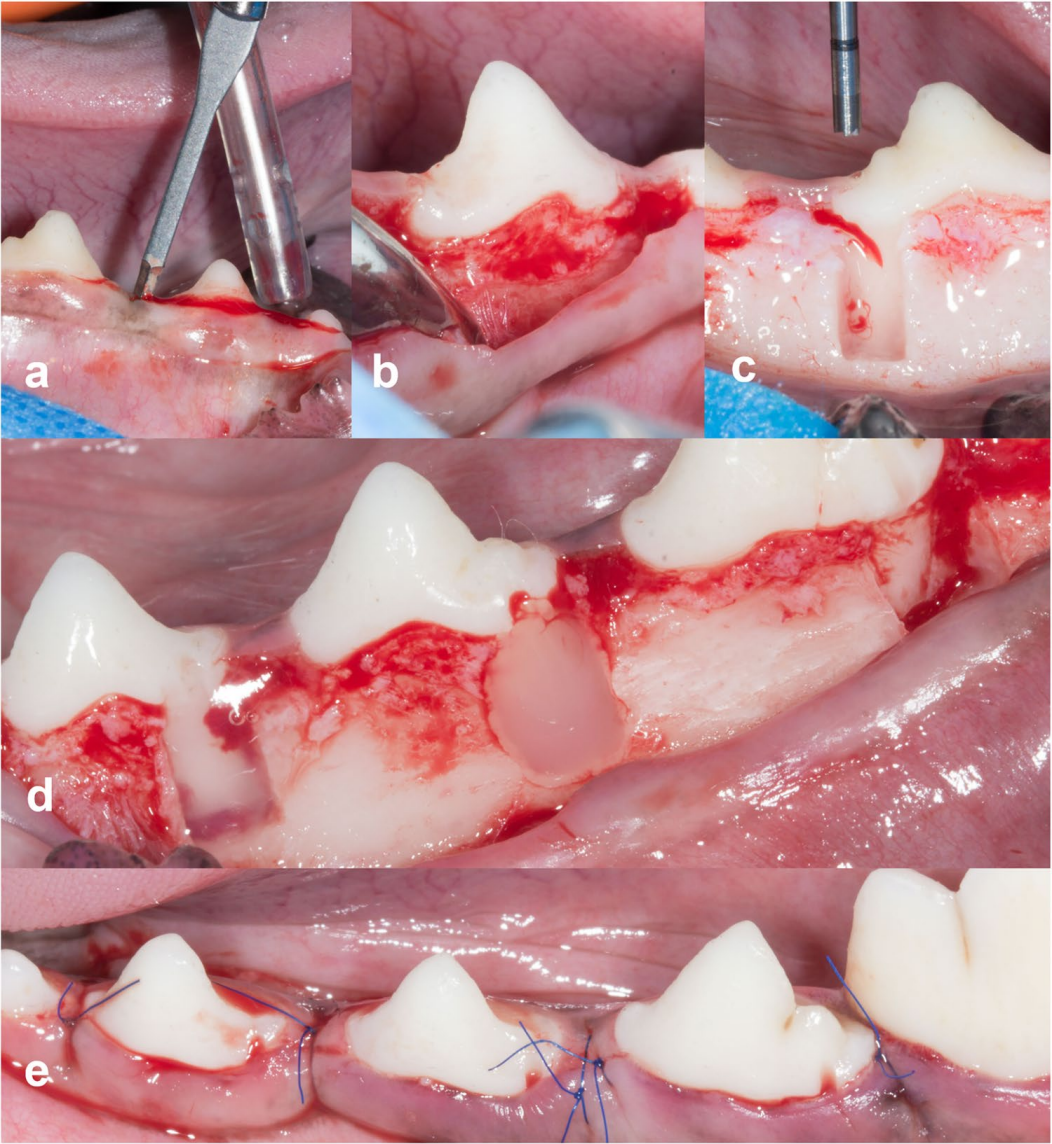
Surgical procedure and treatment: incision (**a**), uncovering the alveolar bone (**b**), creation of standardized defects (5 mm in length and 3 mm in width) (**c**), application of products: test (left), control (middle), sham (right) (**d**); closure of defects (**e**)

The surgical preparation of acute buccal dehiscence-type bone defects was carried out by two surgeons (TW, RJ) in surgical rooms according to the modified method of Hammarström [21, 22]. Shortly, buccal mucoperiosteal flaps were raised after crevicular incision, extending from the first mandibular premolar to the second mandibular molar. After removal of the buccal alveolar bone (standardized defects of 5 mm in length (cervical margin to apical end) and 3 mm in width (Fig. 2)) on the distal root of premolars P2, P3, P4 and molar M1, the periodontal ligament and cementum of these areas were completely removed by means of an end-cutting dental bur. Loupes with a magnification of 5.6 × were used to check for residues of bone. A notch was created on the root surface at the apical end of each bony defect and in the crown indicating the middle of the defect. Treatments were systematically allocated to the four teeth to avoid any potential bias affecting the healing process induced by the tooth location (for details see 28). Usability of the treatment products was evaluated by the surgeons (excellent, good, fair or poor) with respect to ease of application, quality of product’s spreading onto the defect and absence of leakage. The flap was repositioned and securely sutured with a double sling suture per papilla [23] (Dafilon® 6–0, B. Braun / Prolene® 6–0, Ethicon) after treatment application. At the end of the surgery, the dogs were moved to a recovery area, intramuscularly received atipamezole (Alzane®, Zoetis) for awakening if required, monitored for recovery after which each animal was returned to the cage and observed.

Post-operatively, dogs were checked daily for general health and surgical sites were examined daily for ~ 2 weeks (thereafter weekly) recording signs of inflammation. Adverse clinical signs were treated as needed. Furthermore, they received daily anti-microbial prophylaxis with spiramycin and metronidazole for 14 days (Buccoval®, Sogeval) respectively until complete wound healing and suture removal as well as local disinfection with chlorhexidine (Cooper), anti-inflammatory drug (carprofene, Carprodyl®, CEVA) for 5 days following each surgery and analgesia Buprenorphine (Buprecare®, Axience) in the evening following both surgeries. Anti-microbial prophylaxis was also given the day before each tooth scaling until the surgery day as well as analgesia on the day of teeth scaling. Sutures were removed under anaesthesia after 14 days when wounds were completely healed.

Twelve weeks after the first surgery, dogs received analgesia (buprenorphine, Buprecare®, Axience), were anesthetized (tiletamine-zolazepam, Zoletil®, Virbac), intubated, mechanically ventilated and placed on isoflurane inhalant anaesthetics, were heparinized (300 U/kg, intravascular, 25,000 IU Heparin Choay, Sanofi-Aventis) and then euthanized by a lethal injection of a barbiturate (Dolethal®, Vetoquinol).

### Specimen processing procedures

#### Fixation

Each dog’s head was exsanguinated, rinsed with phosphate buffered saline and perfused with 10% neutral buffered formalin (NBF, 4% formaldehyde, CAS 50–00-0). The mandible was carefully cut out with a saw once perfusion was complete. The right and left hemi-mandibles were separated and placed in 10% NBF for 40–48 h for fixation (NBF was exchanged after one day).

Each hemi-mandible was rinsed in a large volume of tap water for 2 h ± 10 min after the fixation period in NBF; water bath was renewed twice. Then the region of interest (teeth P2 to M1 teeth +  ≥ 2 mm) was sawed out and each specimen was kept in Phosphate Buffered Saline (PBS, Sigma) at 4–8 °C until micro-computer-tomography (µ-CT) scanning at NAMSA. µ-CT images were taken as soon as possible as the workflow from euthanasia until placement of specimens into dehydration resp. decalcification solution was considered critical.

#### Histologic preparation and evaluation

Almost all specimen preparation procedures were conducted by NAMSA. In order to embed in poly(methyl methacrylate) (PMMA) and paraffin, the study sites of P2, P3, P4 and M1 were dissected coronally with a diamond saw in two almost equal parts with the help of the vertical notch made in the tooth crown at surgery indicating the middle of the defect. µ-CT images and data were used whenever needed for a better positioning of the cutting axis. The halves were reviewed after dissection and assessed for the decision about which half would undergo PMMA- (main evaluation) respectively paraffin-embedding (28).

#### Decalcification (paraffin-embedding only)

The tooth halves were demineralized using ethylenediaminetetraacetic acid (EDTA, 14%) at 37 ± 5 °C using a shaker for 12–16 weeks. EDTA was changed every three days and tissues were rinsed with PBS to remove released calcium. Decalcification was monitored using needle indentation (every week after the two first weeks) and X-rays for confirmation of uniform decalcification. After decalcification, specimens were dehydrated.

#### Dehydration (PMMA and paraffin specimens), embedding, sectioning and staining

The specimens were dehydrated in a series of graded ethanol solutions and xylene and then embedded. Two serial, transversal buccal-lingual sections of 4–7 µm thickness were obtained from each paraffin block using a microtome (Microm, France) maintaining the anchorage of the tooth integrated to the newly formed soft tissue and alveolar bone and stained with Picrosirius Red (PR) respectively Haematoxylin, Eosin and Safranin (SHE). One transversal bucco-lingual section of ~ 10 µm thickness was obtained from each PMMA block of each study site by laser sectioning and stained with Mc Neal (MN) (LLS ROWIAK LaserLabSolutions GmbH, Hannover, Germany). High resolution scans were taken.

#### Histologic evaluation

Histologic evaluation was performed by a blinded assessor (MH) using microscopes (Olympus CX41RF / Olympus BH-2 and polarization microscope Zeiss MC63 with filters):

1. A general histological description of the specimens
2. Frequency of histologically observed adverse events (evaluation according to ISO 10993–6)
3. Estimation of functional, oriented periodontal ligament (Sharpey’s fibres) relative to the defect in %. This evaluation was controlled using the 4-eyes principle. Consensus in the final assessment was found where a disagreement was identified.

#### µ-CT

**Fig. 3:**
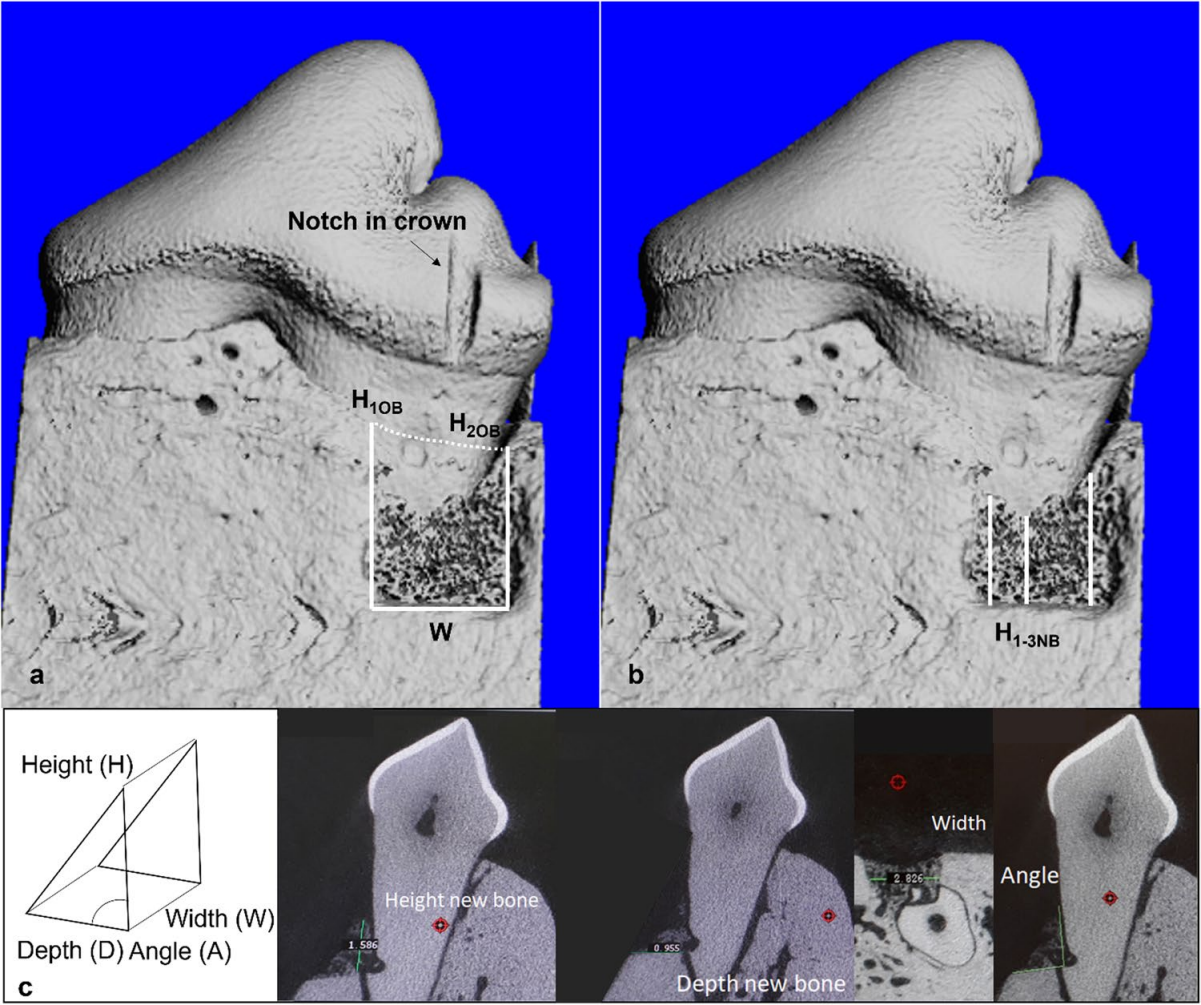
Example of µ-CT measurements and calculations of **a**) original bone level (dog #4, tooth P4, left jaw / 4 W, T2 (top line): two measurements were made—one on the left and one on the right end of the defect (H_1OB_, H_2OB_)—to calculate the average height of original bone level (H_OBA_)), **b**) height of new bone (average (H_NBA_) was calculated from three individual measurements (H_1NB,_ H_2NB,_ H_3NB_)) and **c**) new bone volume that was used for validation of the SCANCO macro (dog #2, tooth P4, left jaw / 4 W, C (bottom line): graphic presentation of the prism and measurement of height, depth, width and angle of the new bone to calculate with the help of the prism formula an approximation of the bone volume)

Each portion of hemi-mandible (P2 to M1) was scanned by cone beam micro-computed tomography (µCT 40, SCANCO, Switzerland) using an energy of 70 kVp, current intensity of 114 μA and settings of 37 mm for field of view and 18 µm isotropic for voxel size. Data was reconstructed, then defects were aligned similarly in the XYZ-plain before the following measurements were conducted on the µ-CT images (using SCANCO Software and macro (mineral density and volume) version 7.0; Fig. 3):

1. Height of the original bone level at both ends of the defect (H_1OB_, H_2OB_): distance between apical notch and coronal extension of original alveolar bone along the root surface. Height measurements of both defect ends were used to calculate average height of original bone level (H_OBA_) which was in the following used as reference for the calculation of relative recovery. Learning for future studies: notch at original bone level should be made in tooth before alveolar bone removal.
2. Height of new bone (H_xNB_; where x are 1–3 measurements depending on the shape of regrowth of the new bone): distance between apical notch and coronal extension of newly formed alveolar bone along the root surface. Average height of new bone (H_NBA_) was calculated from the individual measurements (H_xNB_).
3. Depth of defect (D_D_): distance from the root surface to the buccal end of the alveolar bone at the bottom of the defect.
4. Depth of new bone (D_NB_): distance from the root surface to the buccal end of the new alveolar bone measured at the bottom of the defect.
5. Angle (A) between the line drawn for the measurement of the depth and height.
6. Width (W): mesial-distal length of osseous defect measured at the bottom of the defect.
7. Mineral density of new bone was obtained by the SCANCO macro using the grey value threshold of 185 for 4 weeks and 230 for 12 weeks.
8. Mineral density of sound alveolar bone: the lingual alveolar bone of 7 random teeth (of each dog one) was measured using the same settings as described above for new bone and a mean of 921.5 mg HA/ccm (SD: 34.3) was obtained by calculation.
9. Relative new bone volume with respect to the original alveolar bone volume was assessed using the SCANCO macro with the following settings for threshold grey values: 155 (277 mg HA/cm^3^) for 4 weeks and 200 (415 mg HA/cm^3^) for 12 weeks. The results obtained using the macro were validated against the values received by calculation using the formula of a three-sided prism as the assumption was made that the defect would approximate a three-sided prism. In particular, the following formulas were used:
  $$Defect volume=0.5*\left(DD\right)*\left(HOBA\right)*\mathrm{sin}\left[\left(\frac{Angle^\circ }{180^\circ }\right)*\pi \right]*W$$

respectively

$$New bone volume=0.5*\left(DNB\right)*\left(HNBA\right)*\mathrm{sin}\left[\left(\frac{Angle^\circ }{180^\circ }\right)*\pi \right]*W$$

Furthermore, the obtained results using the macro also underwent a visual plausibility check and were overall found to be representative and valid.

All µ-CT measurements were done blinded and checked by another blinded person.

### Objectives and outcomes

The objectives of this study were to evaluate the performance and safety of a SAP P_11_-4 matrix in comparison to a sham operated control and the clinical gold standard EMD by means of the following endpoints:

#### Performance

- μ-CT endpoints:
  1. Relative recovery of new bone height with respect to the average original alveolar bone height
  2. Relative recovery of the new bone volume with respect to the original alveolar bone volume
  3. Relative recovery of the mineral density of the new bone with respect to the mineral density of sound alveolar bone (normalized to sound dog alveolar bone)

b) Histologic endpoints:
  4. Relative recovery of periodontal ligament’s functionality (Sharpey’s fibres) with respect to the defect
  5. Frequency of downgrowth of junctional epithelium

#### Safety

- Histologic endpoints: gingival recession, ankylosis, root resorption, osteolysis, necrosis, inflammatory cells (evaluation according to ISO 10993-6)
- Clinical endpoints: inflammation and necropsy

### Statistics

#### Sample size

Sample size was not assessed by power calculations due to a lack of existing data. Sample size was for ethical reasons set to an absolute minimum that allowed a first assessment of the main performance parameters. The sample size of 7 dogs was in the range of previous studies, which investigated the gold standard EMD [19, 24, 25].

#### Statistical analysis

Descriptive statistics of all endpoints were computed including the mean, standard deviation (SD) and quartiles for each endpoint and treatment group. Relative recovery of original bone height was further explored by modelling it with two approaches: i) Bayesian and ii) frequentist one. Both models were specified according to the study design, i.e. a repeated measures design with split-plot character. Thus, the dependent variable (relative recovery of original bone height) was modelled as a function of time point, treatment group, interaction between time point and treatment group, dog ID and tooth as independent variables. Moreover, the random intercept of dog ID and jaw side was included in order to account for the repeated measures design. Due to scarce data, special emphasis was laid on checking model assumptions in both approaches. In case of the Bayesian model, convergence criteria (Rhat), posterior distributions and posterior predictive distributions were carefully checked. In case of the linear mixed model used for the frequentist approach, model residuals and diagnostic plots were used for checking model assumptions. The treatment groups were then pairwise compared using marginal means and derived 95% high posterior density intervals and 95% confidence intervals, respectively. All statistical procedures were performed using the statistical software R (2019) [26] including the packages ggplot2 [27], brms [28], loo (2017) [29], lme4 [30], emmeans [31, 32]. No imputations for missing data were used.

## Results

### Usability

The ease of application and the spreading behaviour onto the defects was evaluated as comparable for all treatment groups, namely as excellent/good. The SAPM P_11_-4 test material however, tended to leak more compared to the EMD control.

### Clinical observations

No differences were observed with respect to signs of inflammation between the study groups. Slight/moderate inflammation was observed post-operatively up to maximum 17 days. No abnormalities such as necropsy were observed on the operated sites at any time and for any groups.

### µ-CT and histology

Two sites (T1, 4 W and T2, 4 W) were excluded from analysis as bone wasn’t completely removed at surgery day and for one site (C, 12 W) no sections for histological evaluation were available due to artefacts (supplementary information Fig. S1). For 27/56 defect sites (48%) only the resin section (McNeal) was available for periodontal ligament (PDL) assessment.

### Histological evaluation

Overall, in all groups and at all time points, the crown was intact and covered with an enamel layer. The dentin in the crown and the crown enamel-dentin junction (EDJ) were also unremarkable. Some teeth showed a scalloped surface at the root, which was interpreted as an artefact from handling due to the removal of the alveolar bone and cementum, as were the purposefully placed notches in the crown and at the base of the alveolar bone defect for landmarking. The pulp cavity of all examined teeth showed no histological changes at any time point.

The gingiva showed few to moderate amounts of lymphocytes and plasma cells mostly at the tips and two teeth showed lymph follicle formation in the gingiva (dog #1, left jaw, P4 (T1, 12 W), dog #7, left jaw, P4 (S, 12 W)), both were considered normal immunologic responses due to the normal oral flora. In one case few macrophages and giant cells were observed where hair fragments were left behind at surgery (dog #4, left jaw, M1 (C, 4 W). The gingival epithelium was moderately thickened in all groups and showed deeper rete ridges than normal on the handling side.

In no group and at no time point gingival recession or necrosis was visible, nor were inflammatory cells like neutrophils implying a bacterial infection, eosinophils or cells other than those described above present. Also, no downgrowth of junctional epithelium was detected.

Both, the cementum-enamel junction (CEJ) and the dentin root of the examined teeth were normal except for handling artefacts. The dentin-cementum junction (DCJ) in the area of the defect manipulation was sometimes scalloped but otherwise without histological changes. If the notch placed in the root of the dentinal body was deep, the notch was generally filled with mesenchymal cells, probably derived from the ligament, cementum-like material and with new formed bone. In some defect areas, the cementum was slightly to moderately thicker due to the defect, but overall thinner as the acellular cementum of the body was mostly affected by the manipulation. The cellular cementum of the root was overall normal, but when defects also affected the cellular cementum, then it was thinner. In conclusion, new cementum was altogether observed as a continuous thinner layer in all defects assessed histologically.

The alveolar bone of most teeth showed some to moderate remodelling signs after 4 weeks, especially dogs #1 (right), #3 (right), #4 (left) and #6 (left jaw) showed many osteoblasts and osteoclasts with new bone formation (woven bone).

All but one tooth, which showed only moderate remodelling (dog #1, right jaw, M1 (T1)), demonstrated lamellar bone at 12 weeks, which was well integrated with the original bone. No osteolysis, root resorption or ankylosis was seen.

Around the teeth that showed remodelling after 4 weeks, a moderate to sometimes larger amount of fibrous tissue was visible in a band-like pattern surrounding the outer edge of the bone toward the gingiva to stabilize the defect. The muscles surrounding the teeth showed slight atrophy in many cases. No changes in the cortical bone or neovascularization of the tissue were noted.

**Fig. 4:**
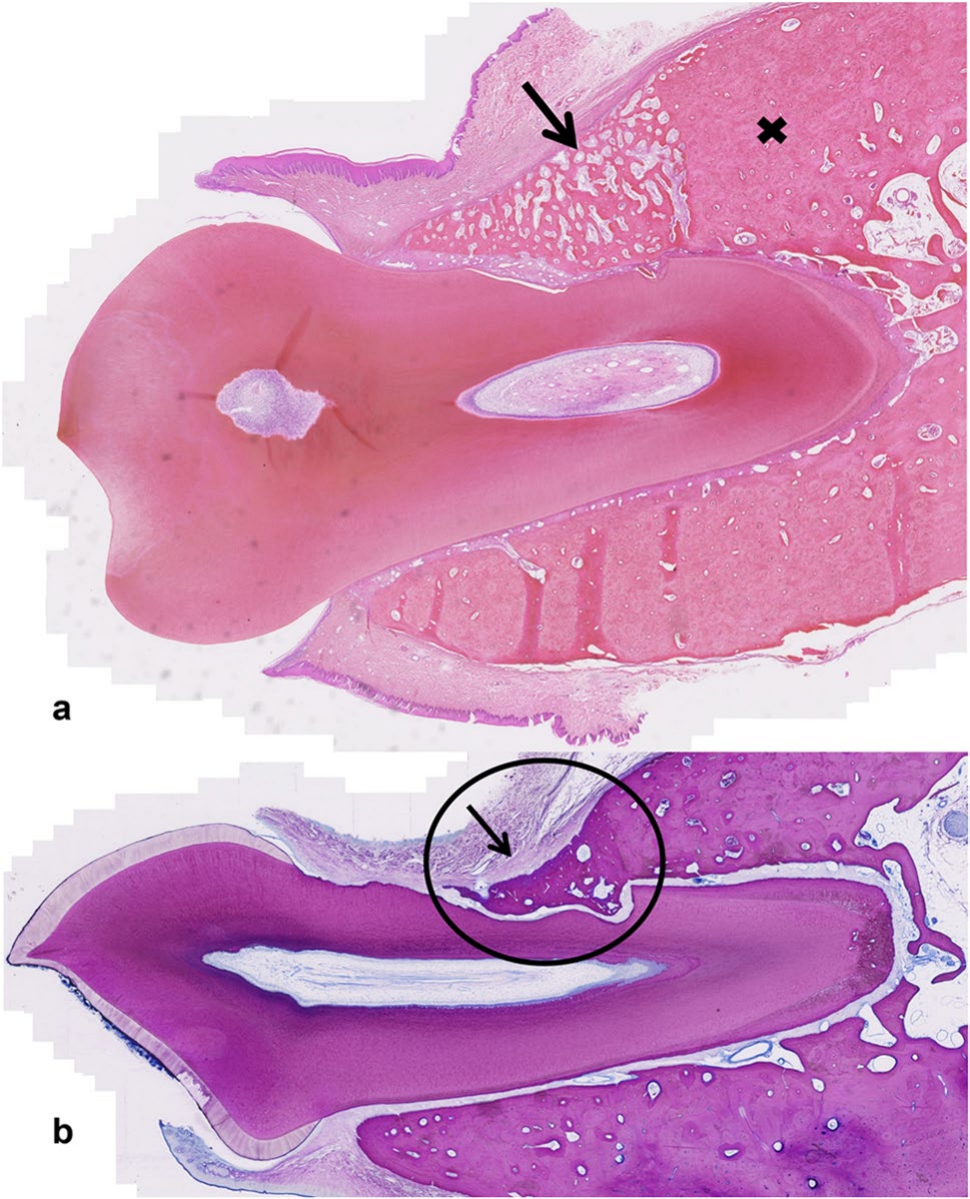
Representative histologic section of a sham-operated site (S) after **a**) 4 weeks (Picrosirus red staining of M1 (dog #1, right jaw) where the arrow shows newly built woven bone and less lamellar bone compared to the adjacent original alveolar bone (cross); relative bone height recovery for this tooth was 0.75 assessed by µ-CT and 25% new functional periodontal ligament (tip) assessed by histologic evaluation), **b**) 12 weeks (Mc Neal staining of P4 (dog #7, left jaw) where the arrow indicates the newly formed fibrous tissue band and the circle the good integrated lamellar bone which is growing into the defect; the defect is covered by a thin rim of acellular cementum; the relative bone height recovery for this tooth was 0.59 and the new functional periodontal ligament was assessed as 100%)

**Fig. 5:**
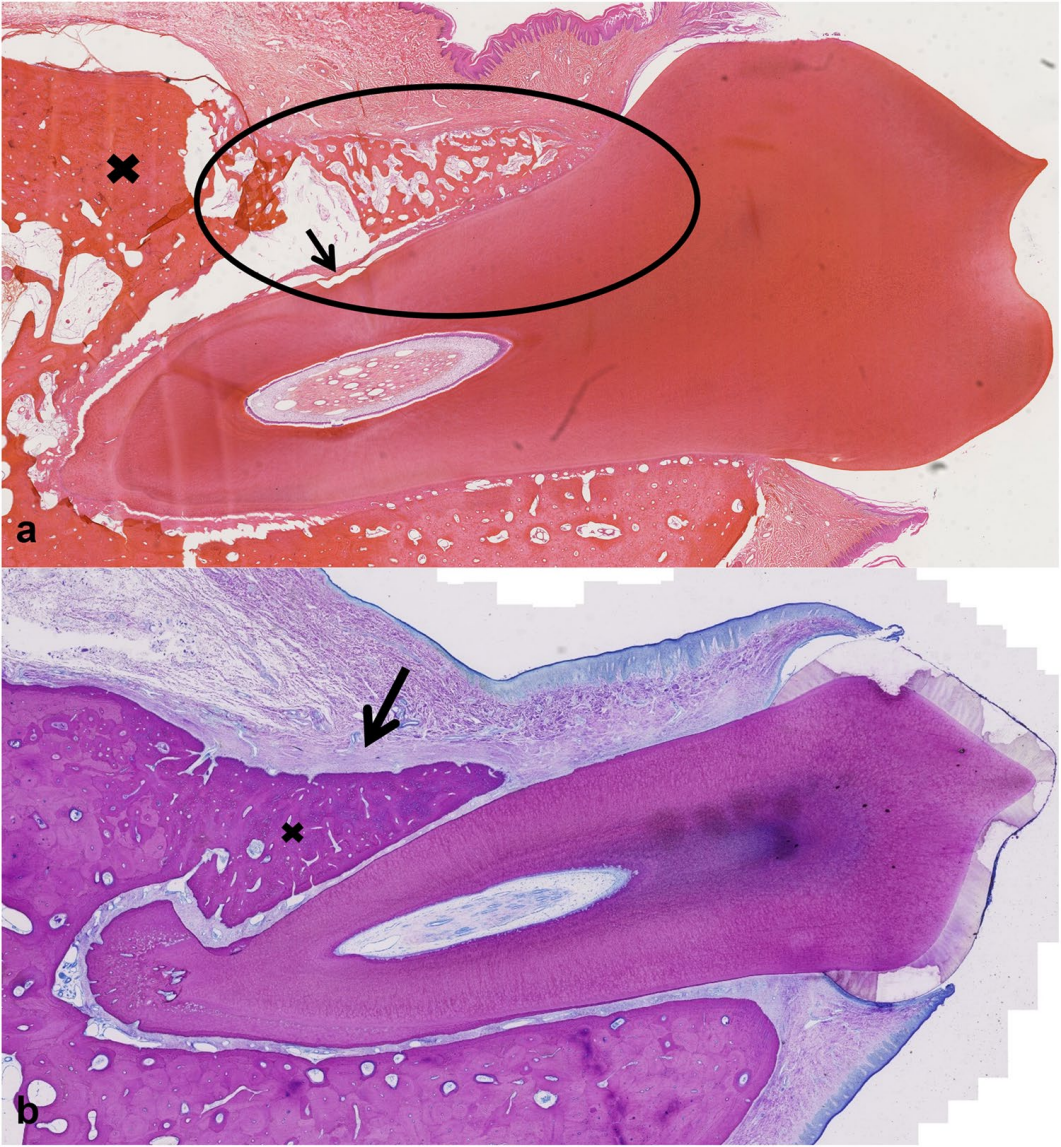
Representative section of a control site (C) after **a**) 4 weeks (Picrosirus red staining of M1 (dog #4, left jaw) where in the circle newly built woven bone is visible, pre-existing alveolar lamellar bone is marked by a cross and the arrow depicts the scalloped tooth surface produced by the bur; relative bone height recovery for this tooth was 1.02 assessed by µ-CT and 20% new functional periodontal ligament (tip) assessed by histologic evaluation), **b**) 12 weeks (Mc Neal staining of P3 (dog #5, right jaw) where the arrow indicates the newly formed thick fibrous tissue band and the cross good integrated newly formed lamellar bone; the relative bone height recovery for this tooth was 0.72, the new functional periodontal ligament was assessed as 90% (10% tip disorientated) and the newly formed cementum was thinner on the defect compared to the sound side)

**Fig. 6:**
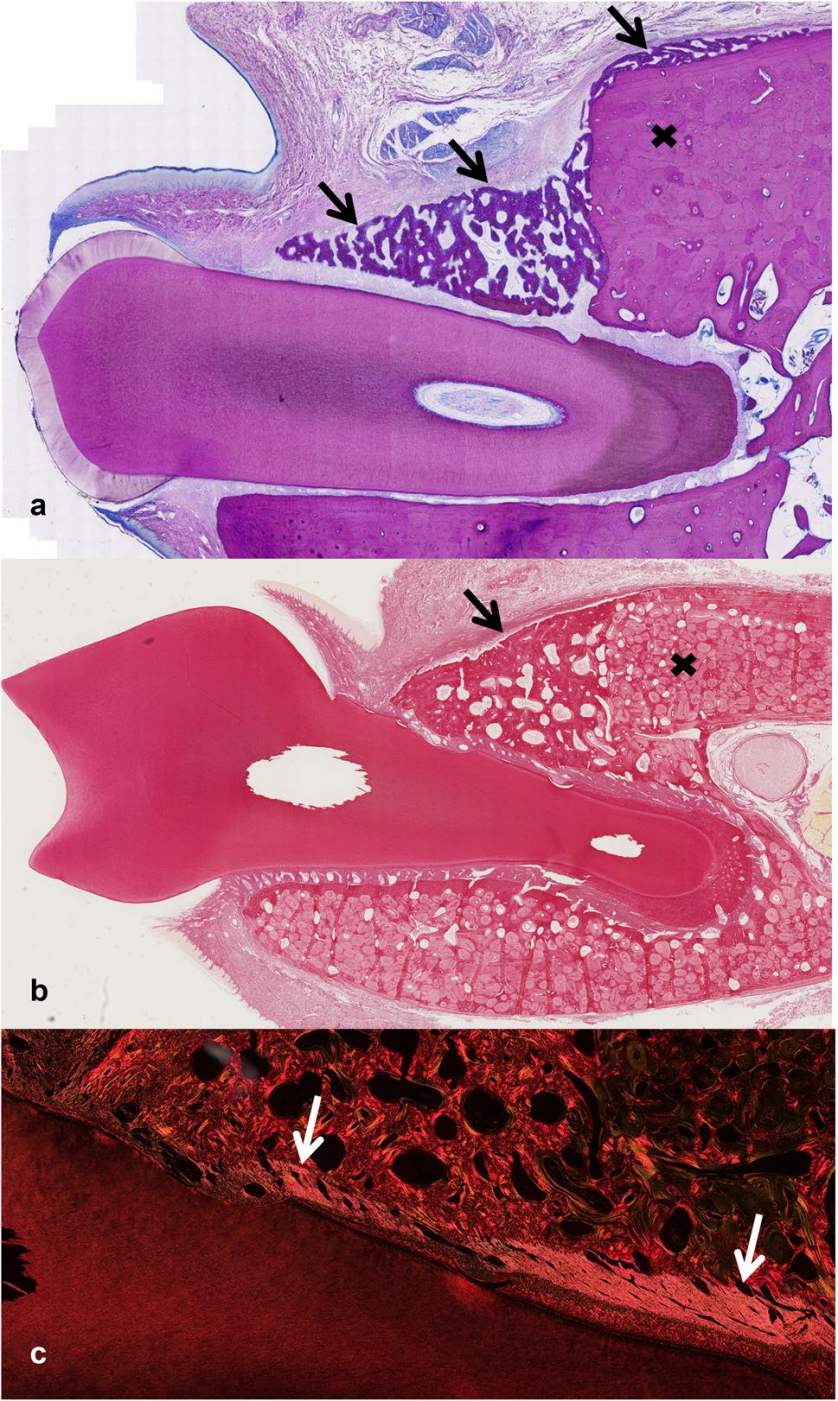
Representative section of a T1 site after **a**) 4 weeks (Mc Neal staining of P2 (dog #1, right jaw) where the arrows show new woven bone formation that overgrowths also pre-existing lamellar alveolar bone (cross); relative bone height recovery for this tooth was 0.80 assessed by µ-CT and 35% new functional periodontal ligament (25% tip, 10% cranial) assessed by histologic evaluation (note: old bone is visible, PDL functionality was assessed regarding new bone only)), **b**) 12 weeks (Picrosirius red staining of M1 (dog # 7, left jaw) which shows good integrated newly formed lamellar bone (arrow) on top of the pre-existing alveolar bone (cross) and the scalloped pattern on the tooth surface due to the bur is good visible; relative bone height recovery for this tooth was 0.94 and the new functional periodontal ligament was assessed as 100%), **c**) inlet of b) showing functional periodontal ligament (arrows) under polarization light

On the defect side, the periodontal ligament that was build was in general slightly thicker. Histological assessment of PDL functionality was difficult. Overall, the pattern was that more non-functional disoriented PDL was observed in 4-week-old specimens and predominantly functionally oriented PDL was seen in the 12-week-old specimens. After 4 weeks, 4/6 (67%) in the T1 group, 1/6 (17%) in the T2 group, 4/7 (57%) in the EMD group (C) and 2/7 (29%) in the sham group (S) showed functional, oriented periodontal ligament indicating a similar performance trend of SAPM P_11_-4 (T1 group) to EMD and a tendency of better performance in comparison to the sham control in the early phase i.e. within the first 4 weeks of healing (T1 group). After 12 weeks 7/7 (100%) in T1, 6/7 (86%) in T2, 6/6 (100%) in the EMD group and 6/7 (86%) in the sham control group showed functional, oriented periodontal ligament (Table 1). Figures 4, 5, 6, to 7 show a representative histological section of each study group.

**Table 1:** Results of the four study groups at the two time points 4 and 12 weeks

Healing period: 4 weeks	Observed values				Estimated values (linear mixed model)				Estimated values (Bayes model)			
Group	S	C	T1	T2	S	C	T1	T2	S	C	T1	T2
Relative recovery of alveolar bone height (µ-CT)					Mean (95% CI)				Mean (95% HPD)			
Mean (SD)	0.65 (0.16)	0.73 (0.16)	0.68 (0.16)	0.65 (0.11)	0.64 (0.54–0.74)	0.71 (0.62–0.81)	0.72 (0.62–0.83)	0.66 (0.55–0.76)	0.64 (0.53- 0.75)	0.71 (0.60- 0.81)	0.72 (0.60–0.84)	0.66 (0.54–0.77)
Median (IQR)	0.67 (0.51–0.78)	0.70 (0.61–0.80)	0.72 (0.58–0.78)	0.70 (0.61–0.71)								
N	7	7	6	6								
Pairwise group comparisons ^a^ (p-value)					ns				ns			
Relative recovery of alveolar bone volume (µ-CT)												
Mean (SD)	0.43 (0.15)	0.53 (0.12)	0.42 (0.07)	0.46 (0.12)								
Median (IQR)	0.43 (0.31–0.56)	0.49 (0.45–0.59)	0.40 (0.39–0.47)	0.45 (0.37–0.50)								
N	7	7	6	6								
Relative recovery of mineral density (µ-CT, normalized to sound dog alveolar bone)												
Mean (SD)	0.64 (0.01)	0.64 (0.02)	0.63 (0.01)	0.64 (0.02)								
Median (IQR)	0.64 (0.62–0.64)	0.65 (0.63–0.66)	0.63 (0.62–0.63)	0.64 (0.63–0.65)								
N	7	7	6	6								
Relative recovery of periodontal ligament’s functionality (%) (histology)												
Mean (SD)	17.9 (37.4)	22.1 (35.8)	43.3 (46.0)	6.7 (16.3)								
Median (IQR)	0 (0–12.5)	10.0 (0–22.5)	30.0 (6.3–83.8)	0 (0–0)								
N	7	7	6	6								
n with function (%)	2 (29%)	4 (57%)	4 (67%)	1 (17%)								
Healing period: 12 weeks	Observed values				Estimated values (linear mixed model)				Estimated values (Bayes model)			
Group	S	C	T1	T2	S	C	T1	T2	S	C	T1	T2
Relative recovery of alveolar bone height (µ-CT)					Mean (95% CI)				Mean (95% HPD)			
Mean (SD)	0.75 (0.18)	0.78 (0.15)	0.78 (0.20)	0.80 (0.21)	0.75 (0.65–0.84)	0.80 (0.71–0.90)	0.76 (0.67–0.86)	0.80 (0.70–0.89)	0.74 (0.64–0.86)	0.80 (0.69–0.91)	0.76 (0.66–0.87)	0.79 (0.69–0.91)
Median (IQR)	0.74 (0.59–0.89)	0.82 (0.71–0.86)	0.80 (0.70–0.92)	0.88 (0.65–0.96)								
N	7	7	7	7								
Pairwise group comparisons ^a^ (p-value)					ns				ns			
Relative recovery of alveolar bone volume (µ-CT)												
Mean (SD)	0.69 (0.18)	0.68 (0.16)	0.67 (0.23)	0.72 (0.12)								
Median (IQR)	0.74 (0.52–0.85)	0.65 (0.64–0.79)	0.70 (0.54–0.83)	0.70 (0.65–0.81)								
N	7	7	7	7								
Relative recovery of mineral density (µ-CT, normalized to sound dog alveolar bone)												
Mean (SD)	0.86 (0.04)	0.85 (0.03)	0.85 (0.03)	0.84 (0.02)								
Median (IQR)	0.86 (0.84–0.89)	0.85 (0.82–0.87)	0.84 (0.84–0.86)	0.85 (0.83–0.85)								
N	7	7	7	7								
Relative recovery of periodontal ligament’s functionality (%) (histology)												
Mean (SD)	79.3 (38.8)	98.3 (4.1)	96.4 (9.5)	78.6 (36.6)								
Median (IQR)	100 (77.5–100)	100 (100–100)	100 (100–100)	100 (75–100)								
N	7	6	7	7								
n with function (%)	6 (86%)	6 (100%)	7 (100%)	6 (86%)								

^a^Comparisons between all groups were made i.e. C-S, C-T1, C-T2, S-T1, S-T2, T1-T2: no statistically significant (ns) difference was found between any pairwise group comparisons using 95% CI and 95% HPD, respectively (supplementary information Table S2 and S4)

Abbreviations: interquartile range (IQR), standard deviation (SD), total number of sites available for evaluation (N), number of sites (n)

**Fig. 7:**
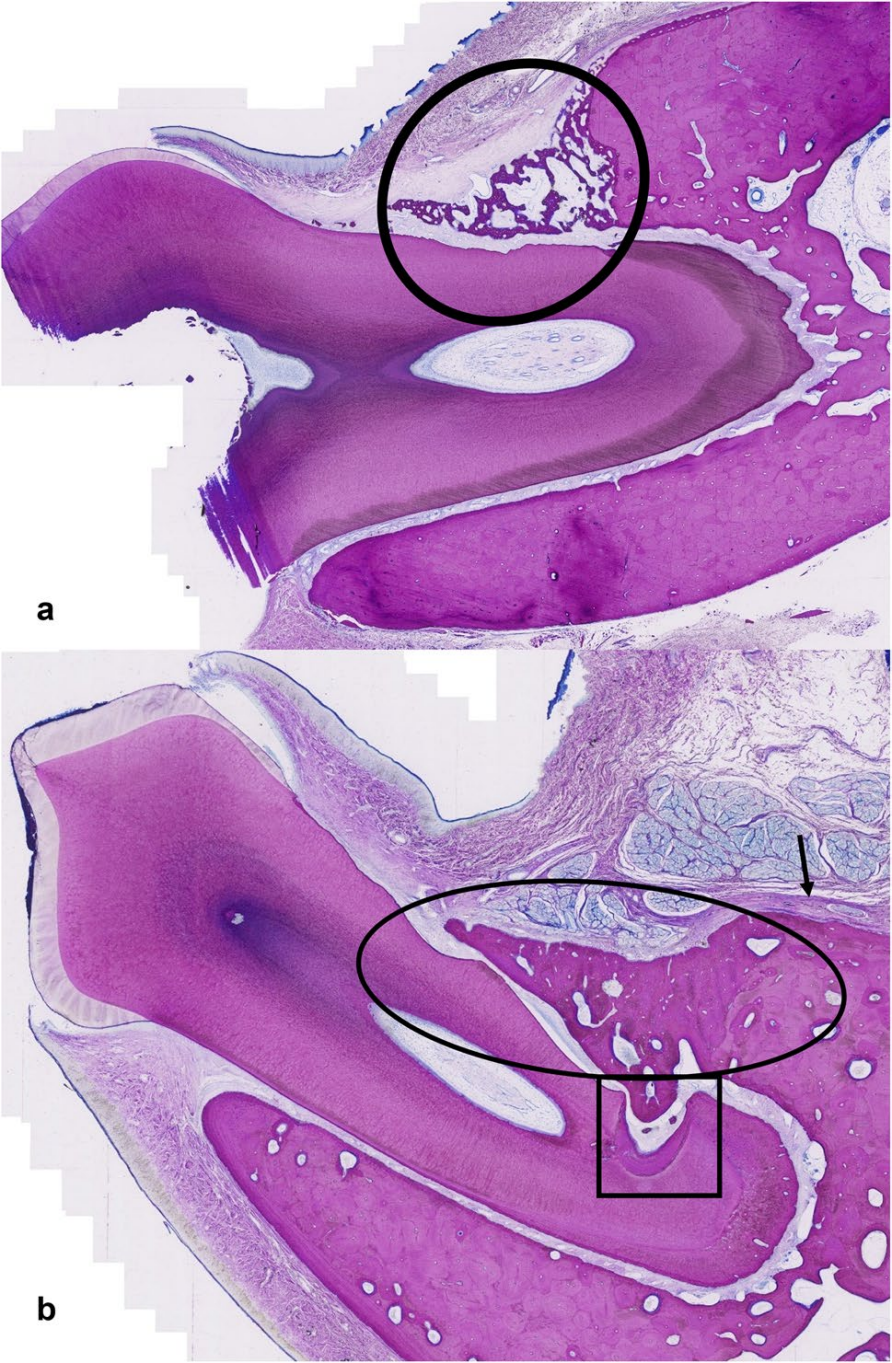
Representative section of a T2 site after **a**) 4 weeks (Mc Neal staining of M1 (dog #2, left jaw) where the circle indicates new woven bone formation on the pre-existing alveolar bone and also a thick fibrous tissue band on the outer surface of the newly formed bone; relative bone height recovery for this tooth was 0.71 assessed by µ-CT and 0% new functional periodontal ligament assessed by histologic evaluation), **b**) 12 weeks (Mc Neal staining of P2 (dog # 7, left jaw) where the square shows a tooth defect covered by a thick layer of acellular cementum and mesenchymal cells as well as lamellar bone filling the tooth defect; the circle indicates good integrated newly formed lamellar bone and the arrow indicates a thick fibrous band crossing between the old and newly formed lamellar alveolar bone; relative bone height recovery for this tooth was 0.75 and the new functional periodontal ligament was assessed as 100%)

In conclusion, no safety or pathological issues due to the treatment were histologically observed in any of the study groups (supplementary information Table S1).

### µ-CT

**Fig. 8:**
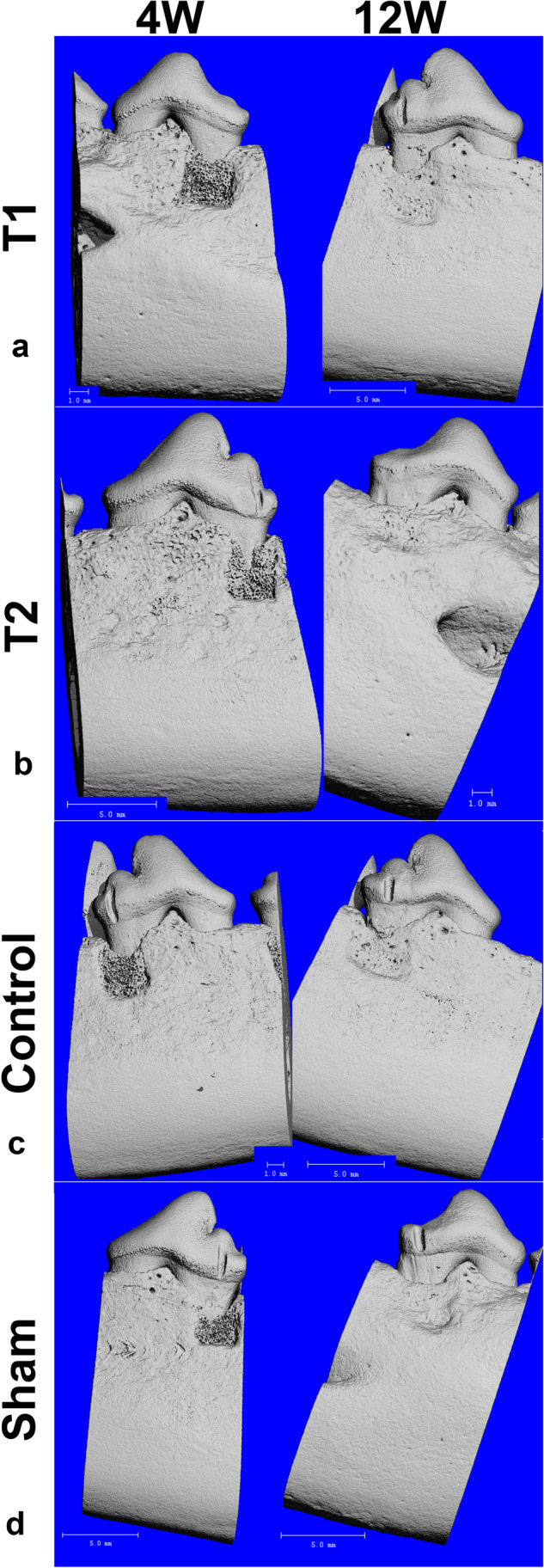
Representative 4 and 12 weeks after treatment µ-CT images for **a**) T1 (4 weeks (left): dog #2, left jaw, P2, relative bone height recovery of 0.70; 12 weeks (right): dog #5, right jaw, P4, relative bone height recovery of 0.72), **b**) T2 (4 weeks (left): dog #4, left jaw, P4, relative bone height recovery of 0.74; 12 weeks (right): dog # 6, right jaw, P2, relative bone height recovery of 0.93), **c**) control (4 weeks (left): dog #1, right jaw, P4, relative bone height recovery of 0.74; 12 weeks (right): dog #6, right jaw, P4, relative bone height recovery of 0.82) and **d**) sham (4 weeks (left): dog # 6, left jaw, P4, relative bone height recovery of 0.57; 12 weeks (right): dog #5, right jaw, P2, relative bone height recovery of 0.83)

The observed median height recovery of the alveolar bone was in the 7 dogs after 4 weeks for T1 (Fig. 8): 72% (IQR: 58–78%), T2: 70% (IQR: 61–71%), EMD (control): 70% (IQR: 61–80%) and sham: 67% (IQR: 51–78%) and after 12 weeks for T1: 80% (IQR: 70–92%), T2: 88% (IQR: 65–96%), EMD: 82% (IQR: 71–86%) and sham: 74% (IQR: 59–89%) indicating similar performance trend of T1 and T2 to EMD and a tendency of better performance in comparison to the sham control.

No statistically significant difference was found among the treatment groups for both time points regarding superiority of our endpoint “relative height recovery of new alveolar bone” regardless of whether the classical frequentist or Bayesian approach was used (Table 1).

Also, no statistically significant difference was found when comparing the two time points within each treatment group (supplementary information Tables S3 and S5). Of note, tooth M1 generally showed a better performance in bone height recovery in comparison to the other teeth.

The observed median bone volume recovery was after 4 weeks for T1: 40% (IQR: 39–47%), T2: 45% (IQR: 37–50%), EMD: 49% (IQR: 45–59%) and sham: 43% (IQR: 31–56%) and after 12 weeks for T1: 70% (IQR: 54–83%), T2: 70% (IQR: 65–81%), EMD: 65% (IQR: 64–79%) and sham: 74% (IQR: 52–85%) indicating a similar performance of T1 and T2 to sham and a tendency of a better performance for EMD in comparison to the other groups after 4 weeks. However, after 12 weeks, the results were the opposite.

The observed median mineral density was comparable between all groups with 0.63 to 0.65 relative to sound alveolar bone after 4 weeks and 0.84 to 0.86 after 12 weeks.

In conclusion, all groups showed overall similar performance results after 4 and 12 weeks of healing regarding new cementum, functionality of newly formed periodontal ligament as well as new bone height, new bone volume and mineral density.

## Discussion

### Interpretation of findings and scientific and clinical implications

The present study focused on the performance and safety evaluation of a novel scaffold consisting of SAPM P_11_-4 for the regeneration of alveolar bone, cementum and functional periodontal ligament in periodontal defects with the goal to restore periodontal attachment.

The most critical parameters to re-establish periodontal attachment are the height of the new alveolar bone as well as the formation of Sharpey’s fibres. In the present study the results of these two parameters taken together indicate a comparable efficacy of the investigational product SAPM P_11_-4 to today’s clinical gold standard EMD and an enhanced efficacy compared to the sham control (periosteum coverage). However, no statistically significant difference among the treatment groups was found after 4 and 12 weeks due to considerable variation in the data resulting from the small sample size. Sample size was based on previous studies investigating EMD and on ethical considerations, as data of SAPM P_11_-4 for the treatment of periodontal defects for a robust sample size calculation was not available. Results of previous studies in animals indicate that EMD supports early stages of wound healing and a difference between EMD and other treatments were observed after two to four weeks [24] but not anymore thereafter [24, 25, 33]. The results of the present study indicate that the SAP P_11_-4 scaffold may support early phases in wound healing similar to EMD. However, to substantiate these findings, further investigations are needed.

Animal-derived products such as EMD have several disadvantages such as the risk of transmitting pathogens or supply problems and ethical issues that may not be acceptable for all patients. Therefore, one of the objectives in the development of new treatment methods is to replace products of animal origin with a synthetic product having similar properties with comparable treatment effect. SAPM P_11_-4 similarly to amelogenin which is part of EMD, self-assembles to form a supramolecular matrix [16]. EMD as well as SAPM P_11_-4 have negatively charged nucleation sites for binding Ca^2+^ ions facilitating biomineralization [34]. The present preclinical study in dogs confirms the results previously obtained by Burke [35] and Saha [34] that SAPM P_11_-4 is a suitable scaffold for bone regeneration based on the regeneration of alveolar bone’s height, volume and mineral density which were comparable to the gold standard EMD. SAPM P_11_-4 may positively influence osteoblasts and their activity enabling enhanced bone growth as a significant higher OPG (osteoprotegrin) / RANKL (Receptor Activator of NF-κB Ligand) ratio was found for SAPM P_11_-4 treated periodontal defects in rats compared to untreated control defects [14]. Furthermore, SAPM P_11_-4 recently was found to enhance proliferation of dental follicle stem cells, osteogenic differentiation and expression of Collagen Type I in vitro [10]. In addition, the present study also supports the results obtained in the first animal study investigating SAPM P_11_-4 in the treatment of periodontal disease [14] where SAPM P_11_-4 treated critical defects showed enhanced formation of Sharpey’s fibres compared to the untreated critical control defects. Favourable cellular interactions between the SAP P_11_-4 scaffold, mediated by fibronectin and other extracellular matrix proteins such as Collagen Type I, III and Fibrillin I absorbed at the scaffold’s surface, and human periodontal ligament fibroblasts led to the observed migration of the periodontal ligament fibroblasts within the SAP P_11_-4 matrix [12, 13] and may contributes to the enhanced formation of periodontal attachment in comparison to the sham-operated control in the present study (T1 group).

Both test groups T1 (pre-treatment with 24% EDTA) and T2 (without a pre-treatment with 24% EDTA) showed successful regeneration and showed similar performance in the present study. Sculean [36] and Mariotti [37] showed in their studies of root conditioners that EDTA (24%, pH 7) does not stimulate periodontal regeneration and therefore, although recommended as pre-treatment procedure in the course of EMD application, may be omitted in the recommendation of SAPM P_11_-4 hydrogel application in the treatment of periodontal disease. In contrast, propylene glycol alginate (PGA) which is the vehicle of the highly hydrophobic EMD has shown significant antimicrobial effects on periodontal pathogens [38–40].

No adverse events such as gingival recession, ankylosis, root resorption, osteolysis, necrosis or fatty infiltrate were observed in the present study. In the study performed by Burke, no specific antibodies against SAPM P_11_-4 were detected in the serum of rabbits 3, 10, 28 and 84 days after SAPM P_11_-4 hydrogel application (30 mg/mL, 0.3 mg SAP P_11_-4) on critical defects in the rabbits’ calvaria. Similar to the present study, there was also no necrosis, inflammation, foreign body reaction, predisposition to infections or any other obvious local, pathological tissue responses observed during the study period of 12 weeks [35]. Both studies confirm the safety of SAPM P_11_-4 hydrogels in soft and/or hard tissue regeneration.

Taken together, SAPM P_11_-4 is a potential and promising candidate as a substitute of xenograft-based products as it is completely synthetically manufactured. Furthermore, this study proofed SAPM P_11_-4 to be a safe product without any cytotoxic effects for the application in periodontal disease. It exhibits modifiable properties like its stiffness [11] that allows the SAPM P_11_-4 hydrogel to be formulated in different forms for different indications such as gingivitis or periodontitis, for solely soft or soft-hard tissue regeneration. It may be used in addition as a delivery and release system of anti-microbial agents as it proofed suitable for this purpose recently [10].

The chosen acute dehiscence model is not without limitation as spontaneous regeneration is possible [18]. In addition, the periosteum that functions as a natural membrane over the acute defects, could not be removed during acute defect creation. The periosteum was shown to exhibit regenerative potential [41, 42] which may explain the considerable regeneration found in the sham control sites. In natural periodontal defects caused by chronic inflammatory processes, the periosteum is not present to support regeneration. As an alternative model, a combined acute-chronic dehiscence model could have been chosen [25, 33] where ligatures are placed for 8–12 weeks around teeth for bacteria colonization and calculus accumulation resulting in the creation of naturally induced periodontal defects which are surgically corrected after ligature removal. Both procedures produce standardized, reproducible defects. However, we opted for the acute model because the burden on the dogs is reduced. An additional preclinical trial with chronic defects would be helpful to assess the full potential of SAPM P_11_-4 in comparison to EMD and sham treatments.

The strength of our study was its blinded evaluation (µ-CT and histology), so any assessment bias can be excluded. However, histologic evaluation by nature is subjective and as functionality of the PDL (semi-quantitative) needed to be assessed in 48% of sites on Mc Neal stained sections only, evaluation bias can’t be excluded in this parameter. Hence, PDL results should be interpreted with caution.

Safety of SAPM P_11_-4 in the treatment of periodontal disease could be demonstrated in this pre-clinical study, therefore we consider that all safety requirements are fulfilled to proceed with a clinical study in humans to evaluate the treatment efficacy of SAPM P_11_-4 in the target population and periodontal defects of natural origin. Although periodontal defects in dogs are closely related to periodontal disease in humans in terms of etiopathology [18] an evaluation by means of a clinical study in humans is required for efficacy confirmation. We propose a clinical study in humans as a next step due to ethical considerations of performing animal studies and to replace and reduce the number of animals in research according to the principle of the R3s (reduction, replacement, and refinement) [43].

## Conclusion

Within the limitations of this proof-of-concept study, the results suggest a regenerative potential of a SAPM P_11_-4 hydrogel for the use in periodontal surgery to promote periodontal regeneration. No adverse effects or safety issues which might affect clinical usage of the product were observed and therefore a controlled, randomised clinical study in humans should be performed as a next step to evaluate the regenerative efficacy and safety of SAPM P_11_-4 in the target population and defects of natural origin. The synthetic SAPM P_11_-4 may offer a safe alternative to the animal-derived product EMD or periosteum coverage in the near future.

## Supplementary Information

Below is the link to the electronic supplementary material.

Supplementary file1 (PDF 681 KB)
