# Supplementary material for: Efficacy and safety of P11-4 for the treatment of periodontal defects in dogs
Source: Clin Oral Investig. 2022 Jan 10;26(3):3151–66. doi: 10.1007/s00784-021-04297-6 (PMC8898238; doi:10.1007/s00784-021-04297-6)
Supplement: Supplementary file 1 — Supplementary file1 (PDF 681 KB) [file 784_2021_4297_MOESM1_ESM.pdf]

SUPPLEMENTARY INFORMATION

Efficacy and Safety of P_{11}-4 for the treatment of periodontal defects in Dogs

Claudine Bommer[1], Tobias Waller[2], Monika Hilbe[3], Daniel Wiedemeier[4], Nina Meyer[5], Stephanie Mathes[5], Ronald Jung[2]

Affiliations of the authors
[1]credentis ag, Dorfstrasse 69, 5210 Windisch, Switzerland
[2]University of Zurich, Clinic for Reconstructive Dentistry, Plattenstrasse 11, 8032 Zurich, Switzerland
[3]Laboratory for Animal Model Pathology (LAMP), University of Zurich, Institute of Veterinary Pathology, Winterthurerstrasse 268, 8057 Zurich, Switzerland
[4]University of Zurich, Center of Dental Medicine, Statistical Services, Plattenstrasse 11, 8032 Zurich, Switzerland
[5]Zurich University of Applied Sciences, Department for Chemistry and Biotechnology, 8820 Wädenswil, Switzerland

Corresponding author: Ronald Jung, email: ronald.jung@zzm.uzh.ch, phone: +41 44 634 32 60

Allocation generation

The allocation list was generated by random draw with the following conditions:

- each treatment was available twice in a same tooth (P2, P3, P4 or M1) so as to guarantee that each tooth received each treatment once or twice.
- the treatment that was drawn only once for the previous tooth was systematically used for the following tooth (for example, sham treatment was drawn only once for P2 for the first surgery and was immediately selected for P3) so as to get a total of 7 teeth treated with each treatment.
- when a treatment was already given in a dog and was drawn again, it was replaced until drawing a new treatment. This avoided to get a same treatment several times in a same dog and to reduce inter individual variability.
- for the treatment of the last tooth of each hemi-mandible, the treatment was selected purposely so as to give a treatment that was not already given.

Supplementary Figures

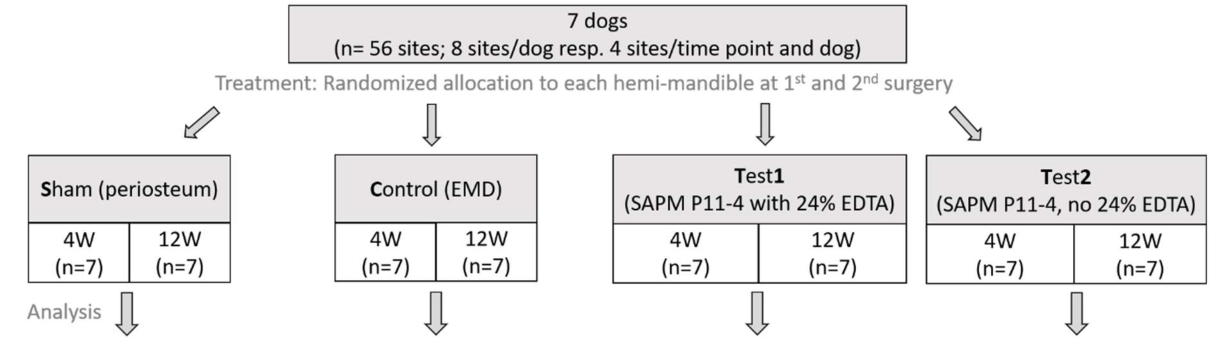

Sham (periosteum)		
N (%)	4W	12W
µ-CT	7/7 (100%)	7/7 (100%)
Histology		
MN	7/7 (100%)	5/7 (71%)
SHE	3/7 (43%)	4/7 (57%)
PR	3/7 (43%)	4/7 (57%)

Control (EMD)		
N (%)	4W	12W
µ-CT	7/7 (100%)	7/7 (100%)
Histology		
MN	6/7 (86%)	6/7# (86%)
SHE	5/7 (71%)	1/7# (14%)
PR	4/7 (57%)	1/7# (14%)

Test1 (with 24% EDTA)		
N (%)	4W	12W
µ-CT	6/7* (86%)	7/7 (100%)
Histology		
MN	6/7* (86%)	6/7 (86%)
SHE	4/7* (57%)	5/7 (71%)
PR	3/7* (43%)	3/7 (43%)

Test2 (no 24% EDTA)		
N (%)	4W	12W
µ-CT	6/7* (86%)	7/7 (100%)
Histology		
MN	5/7* (71%)	7/7 (100%)
SHE	2/7* (29%)	2/7 (29%)
PR	2/7* (29%)	3/7 (43%)

Fig. S1: Overview of available data for evaluation. *Bone was not completely removed during surgery for one site (T1, 4W and T2, 4W) therefore only 6 sites instead of 7 sites were in total available for analysis. [#] For one site (C, 12W) were no sections at all for histologic evaluation available (artefacts). Abbreviations: healing period of 4 weeks (4W) respectively 12 weeks (12W), histologic staining with Mc Neal (MN), with Haematoxylin, Eosin and Safranin (SHE) and Picrosirius Red (PR)

Supplementary Tables

Supplementary Table S1: Summary of safety results assessed by histology

Parameter	Number in which event was observed resp. comment	Mean score (SD)[a]
Epithelial ingrowth	0	0 (0)
Gingival recession	0	0 (0)
Ankylosis	0	0 (0)
Root resorption	0	0 (0)
Osteolysis	0	0 (0)
Necrosis	0	0 (0)
Fatty infiltrate	0	0 (0)
Neovascularization	Normal (as in healing process common; not pathologic)	NA
Fibrosis (according to ISO 10993-6)	In all available sections, a thicker stratum fibrosum was observed on the outer edge of bone which is a normal reaction to stabilize defect/tissue	2.0 (0.6)
Atrophy	Always observed in the area of fibrosis, normal reactive process due to fibrosis	1.2 (0.4)
Inflammation	No pathologic accumulation of polymorphonuclear cells (neutrophils) was observed.<br>Lymphocytes and plasma cells were observed as<br>as expected for the oral cavity.<br>Macrophages and/or giant cells (osteoclasts) were observed, as expected, where bone fragments/hairs had been left behind by surgery.	0 (0)

[a] Evaluation was done using the following scores: 0 (none), 1 (slight), 2 (moderate), 3 (marked), 4 (severe)

Bayesian model

Supplementary Table S2: Comparisons of relative recovery of alveolar bone height between study groups for 4 and 12 weeks using the Bayesian model

Relative recovery of alveolar bone height			
Contrasts	Estimate median	95% Lower HPD	95% Upper HPD
4 weeks			
C-S	0.07	-0.04	0.18
C-T1	-0.01	-0.13	0.11
C-T2	0.06	-0.06	0.17
S-T1	-0.08	-0.20	0.04
S-T2	-0.01	-0.13	0.10
T1-T2	0.07	-0.06	0.19
12 weeks			
C-S	0.06	-0.06	0.16
C-T1	0.04	-0.07	0.15
C-T2	0.01	-0.10	0.12
S-T1	-0.02	-0.13	0.09
S-T2	-0.05	-0.17	0.06
T1-T2	-0.03	-0.14	0.08

Supplementary Table S3: Comparison of relative recovery of alveolar bone height between 4 weeks and 12 weeks using the Bayesian model

Relative recovery of alveolar bone height				
Group	Contrasts	Estimate median	95% Lower HPD	95% Upper HPD
C	4-12W	-0.09	-0.25	0.06
S	4-12W	-0.10	-0.26	0.05
T1	4-12W	-0.04	-0.20	0.12
T2	4-12W	-0.14	-0.30	0.02

Frequentist model

Supplementary Table S4: Comparisons of relative recovery of alveolar bone height between study groups for 4 and 12 weeks using the linear mixed model

Relative recovery of alveolar bone height [a]			
Contrasts	Estimate mean	SE	p-value
4 weeks			
C-S	0.07	0.05	0.54
C-T1	-0.01	0.06	1.00
C-T2	0.06	0.06	0.74
S-T1	-0.08	0.06	0.47
S-T2	-0.01	0.06	0.99
T1-T2	0.07	0.06	0.65
12 weeks			
C-S	0.06	0.05	0.72
C-T1	0.04	0.05	0.89
C-T2	0.01	0.05	1.00
S-T1	-0.02	0.05	0.99
S-T2	-0.05	0.05	0.79
T1-T2	-0.03	0.05	0.94

[a] Results are averaged over the levels of: dog, tooth. Degrees-of-freedom method: Kenward-Roger. p-value adjusted: tukey method for comparing a family of 4 estimates.

Supplementary Table S5: Comparison of relative recovery of alveolar bone height between 4 weeks and 12 weeks using the linear mixed model

Relative recovery of alveolar bone height [a]				
Group	Contrasts	Estimate mean	SE	p-value
C	4-12W	-0.09	0.07	0.19
S	4-12W	-0.11	0.07	0.12
T1	4-12W	-0.04	0.07	0.56
T2	4-12W	-0.14	0.07	0.052

[a] Results are averaged over the levels of: dog, tooth. Degrees-of-freedom method: Kenward-Roger
